# Upregulation of selected HERVW loci in multiple sclerosis

**DOI:** 10.1186/s13100-021-00243-1

**Published:** 2021-06-29

**Authors:** Sofía Macías-Redondo, Mark Strunk, Alberto Cebollada-Solanas, José-Ramón Ara, Jesús Martín, Jon Schoorlemmer

**Affiliations:** 1 Instituto Aragonés de Ciencias de la Salud (IACS), c/Juan Bosco 13, 50009 Zaragoza, Spain; 2 Sequencing and Functional Genomics, Aragon Biomedical Research Center (CIBA), Instituto Aragonés de Ciencias de la Salud (IACS), Zaragoza, Spain; 3 Aragon Biomedical Research Center (CIBA), Instituto Aragonés de Ciencias de la Salud (IACS), Unidad de Biocomputación, Zaragoza, Spain; 4 Instituto de Investigación Sanitaria de Aragón (IIS Aragón), Zaragoza, Spain; 5 Department of Neurology, University Hospital Miguel Servet, Zaragoza, Spain; 6 ARAID Foundation, Avda. de Ranillas 1-D, 50018 Zaragoza, Spain; 7 Placental pathophysiology and fetal programming research group del IISA, c/Juan Bosco 13, 50009 Zaragoza, Spain

## Abstract and Introduction

Human endogenous retrovirus (HERV) are the present day versions of retroviral germline infections that have occured millions of years ago, which occupy about 8 % of the genome [1]. While they are mostly replication deficient, they are known to express RNA and protein [2] during particular developmental stages, or as a response to aging [3], inflammation and a wide range of pathologies [4]. A human retrovirus discovered in Multiple Sclerosis (MS) patients [5], turned out to be the prototype of a novel HERV family referred to as HERVW [6]. The HERVW family consists of 213 elements, 12 out of which are complete proviral copies with intact LTRs [7]. Increased expression of HERVW in peripheral blood mononuclear cells (PBMCs) has been repeatedly associated with MS, and the presence of HERVW protein or elevated RNA transcription has been correlated with disease activity [8–10]. While a contribution of HERVW-encoded proteins to brain disease is suggested by their presence in MS-associated brain lesions, expression in peripheral organs may be involved in the disease process through cytokine-induced damage to the blood brain barrier and subsequent infiltration of monocytes. Alterations in peripheral expression may also serve as a useful and practical marker for the diagnostics of this CNS disease. Therefore, we quantified overall HERVW levels and identified individual HERVW *loci* actually transcribed in PBMCs. Analysis was carried out in patients diagnosed with Clinically Isolated Syndrome (CIS), a precursor to MS, defined by a single episode of neurologic symptoms lasting at least 24 h. CIS is an indicator of future development of MS, as 60 % of the people diagnosed with CIS develop MS [11]. These patients potentially represent the earliest stage of MS routinely available for clinical analysis. We undertook a Next Generation Sequencing (NGS)-based analysis of transcripts amplified from cDNA obtained from patients with CIS and samples from healthy controls. Data presented from this pilot experiment indicate that the relative frequency of specific HERVW copies is altered in PBMC of CIS patients, even in the absence of overall HERVW overexpression. Such altered frequency appears to be derived from less abundantly transcribed but potentially MS-related HERVW loci.

## Methods

### Patients (Table 1)

The local ethics committee (CEICA) approved the study protocol (CP - CI PI14/0021 dated 26/02/2014; modified on 25/10/2017), and patients provided written informed consent (protocolo y información para el paciente v2 de 29/12/2013). Blood samples were collected from MS patients and healthy controls from the Neurology Department of Miguel Servet University Hospital (Zaragoza, Spain). Whole fresh blood was drawn into vacutainer tubes (Becton Dickinson Vacutainer) containing EDTA. Within 24 h, PBMC were isolated as previously described [12, 13].

**Table 1:**
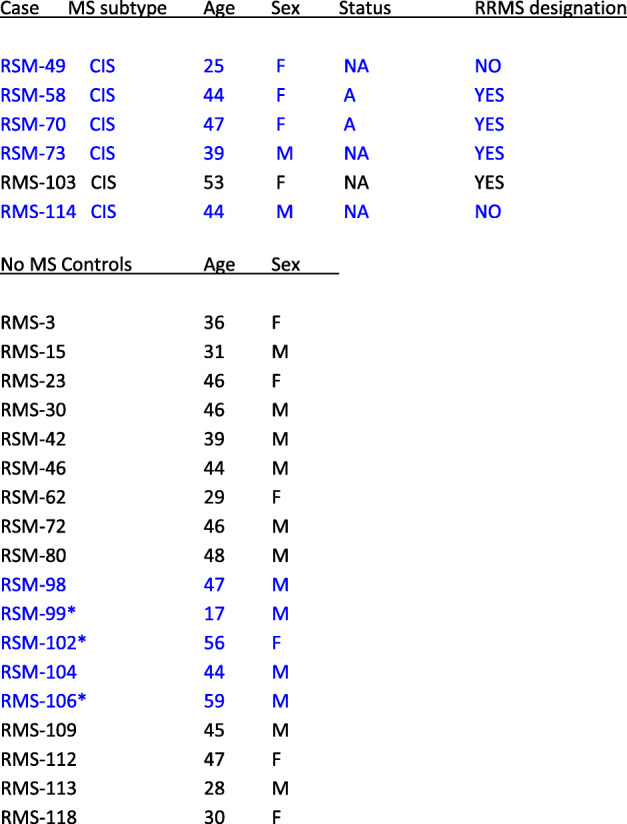
Clinical features of MS patients included in this study

Clinical data of patients whose PBMCs were analyzed for HERVW expression. Median ages for both patients and controles groups were 44 years (mean and SEM are 42,0 +/- 4,25 and 40,4 +/- 1,94 for patient and control groups, respectively)

A/NA status refers to active and non-active patients respectively. Posterior progession towards MS diagnosis (RRMS) is indicated for all CIS cases. Samples analyzed by NGS are marked in blue. median ages in these groups are 44 year for patients and 47 years for controls. * indicates samples only analyzed by NGS

### Expression analysis and PCR

RNA isolation and random-primed cDNA synthesis [14] was carried out as described before. HERVW *ENV* levels were determined by triplicate qPCR assays as described [14, 15]. For the identification and localization of transcribed HERVW *loci*, cDNA was amplified employing the external primers of an established PCR assay for HERVW *ENV* [15]. Products were purified and subjected to NGS analysis.

### NGS analysis

Library preparation and sequencing was carried out using the IonTorrent technology workflow on an Ion Torrent S5XL platform using an Ion 530 chip. Resulting reads were mapped to the human reference genome (version hg19) using strict criteria to maximize mapping differences between different HERVW copies. Relative frequencies were calculated as the number of reads mapping to an individual HERVW ENV element relative to the total number of reads. Details in Suppl. Methods.

### Statistical analysis

SPSS software was used for all analyses and graphs (Version 15.0). Normality and statistical significance of differences were assessed using specific tests. Data were further analyzed using the DESeq2 package [16] to correct *p* values for multiple testing (False Discovery Rate < 0.05).

More detailed information is available in Suppl. M&M.

## Results

**Fig. 1:**
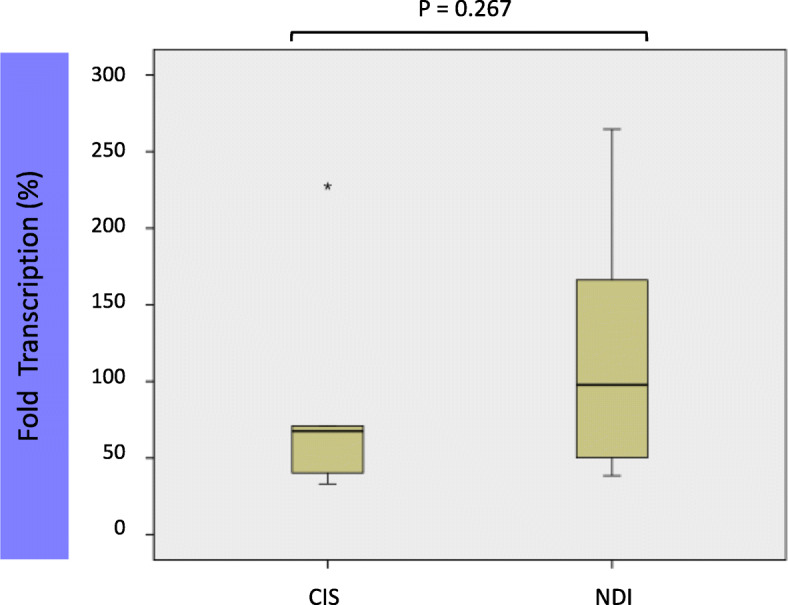
HERVW expression levels in CIS patients. Expression analysis of HERVW ENV levels in CIS (*n*=6) patients and Non-diseased individuals (*n*=15) or NDI (controls) was analyzed by qPCR. Results were normalized using GAPDH as a reference gene, and are represented as the fold expression compared to the median expression level in controls (recalculated as a percentage). U-Mann-Whitney test; *P* = 0.267

We carried out HERVW *ENV* expression analyses using an optimized assay described by Mameli et al. [15]. No significantly increased expression of HERVW was detected in a small cohort of CIS patients (*n* = 6) compared to age-matched controls (*n* = 15)(U-Mann-Whitney *p* = 0.267) (Fig. 1). Results were not skewed by the use of *GAPDH* as a reference gene (Fig. 1), as comparison with *RPL19* and *HSDA* reference genes (Table S1 and Suppl Figure 1) showed that there is no statistical difference between the use of either *GAPDH* or the mean of the three genes (Welch´s t-test; *p* < 0.05).

**Fig. 2:**
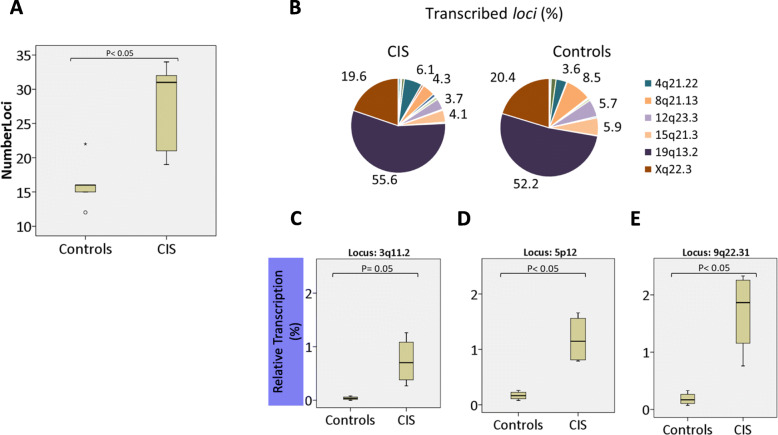
NGS analysis of transcribed HERVW ENV copies in PBMCs. A specific MSRV ENV PCR assay [15] was applied to random primed cDNA from CIS patients (*n*=5) and Controls (*n*=5) and products were sequenced by NGS. For each sample, the relative frequency (%) of reads mapping to individual HERVW ENV copies was calculated relative to the total number of mapped reads. **A**) The total number of transcribed HERVW ENV copies identified (*p*=0.018; student’s T test). **B**) Individual HERVW ENV copies and the mean relative frequency (%) of reads mapping to each copy is represented in pie charts. The most abundantly transcribed copies are indicated. **C**-**E**) The median relative frequency (%) of reads mapped to the individual HERV-W ENV copies in CIS patients or controls is indicated

In the absence of increased overall expression levels of HERVW in CIS samples, we wondered whether specific copies of HERVW (Table S2) might be differentially expressed. We performed NGS analysis to identify individual HERVW copies with altered expression in PBMC from CIS patients (*n* = 5) and controls (*n* = 5). Reads obtained (70,694 ± 24,812 per sample; 25,286–136,704) were mapped to the human genome. Once assigned to unique genomic locations, reads corresponding to 39 HERVW ENV *loci* were extracted (Table S3 and Table 2). As expected, > 99.85 % of mapped reads correspond to the 39 *loci* analyzed (data not shown). The resulting data showed that reads obtained from CIS patients mapped to a significant higher number of different HERVW ENV loci (31 ± 13), compared to those obtained from controls (16 ± 5.5) (t-student; *p* = 0.018) (Fig. 2 A). Over 70 % of the reads mapped to either of two loci: 19q13.2, Xq22.3. Extending the range, reads mapped with high frequency (> 3.6 % of total reads/*locus*) to a limited number of *loci*, in particular to HERVW ENV copies located on chromosomes 19q13.2, Xq22.3, 8q21.11, 15q21.3, 12q23.3 and 4q21.22 (Fig. 2 B). We found no significant differences between CIS patients and controls in the relative frequency of reads mapping to these *loci* (Fig. 2 B; Table 2).

**Table 2:**
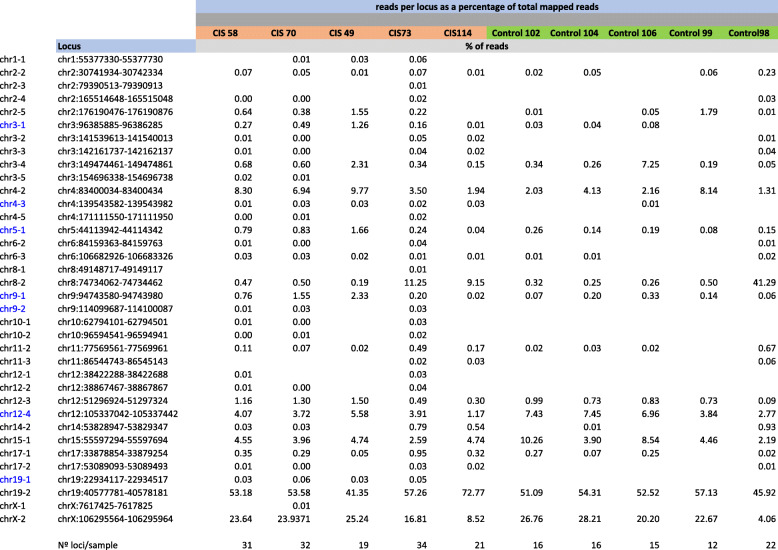
Percentages of reads mapped to individual HERVW *loci*

Mapped reads (Table S3), were recalculated as the number of reads mapping to an individual HERVW ENV element relative to the total number of reads, and represented as a percentage. The HERVW *loci* to which an increased number of reads mapped in CIS patients are indicated in blue

Lower numbers of reads mapped to the remaining 33 loci, with relative frequencies ranging from 0,01 to 1,66 %. We found differences between CIS patients and controls in the relative frequency of reads mapping to several of these. Reads mapping to a subset of HERVW loci, including copy_chr3-1, copy_chr4-3, copy_chr5-1, copy_chr9-1, copy_chr9-2, copy_chr12-4 and copy_chr19-1 differed between CIS patients and controls (Table 3; Fig. 2 C-E). The relative frequency (ranging from 0,3 − 2,95 %) of reads mapping to these loci showed a > 7 fold increase in CIS patients compared to controls (Table 3). When corrected for multi-locus analysis, significant differential expression (*p* < 0.05; FDR < 0.05) of five of these loci loci was evident (Table 3), with significant increases in expression of HERVW copies 3q11.21, 4q31.1, 9q31.3 and 19p12 and a significant decrease in 12q23.3.

**Table 3:** HERVW copies differentially expressed in CIS patients

HERVW Copy	Repeatmasker name	Reads in CIS	Reads in Controls	Fold increase	*P* value
chr 3-1	3q11.2	2221	124	14,76	0,0040
chr 4-3	4q31.1	92	6	12,64	0,0333
chr 5-1	5p12	3999	562	5,86	0,27
chr 9-1	9q22.31	5678	614	9,2	0,12
chr 9-2	9q31.3	81	1	> 20	0,0333
chr12-4	12q23.3	18962	20812	-1,33	0,0015
chr19-1	19p12	192	1	> 20	0,0015

The table lists several HERVW copies, using references from Tables S2-S3. Names used in RepeatMasker are also indicated. The sum of mapped reads in samples from five CIS patients or five controls is listed, as well as the resulting fold change in CIS versus control. The *P* value for the difference between NDI and CIS samples was adjusted for multiple testing (FDR <0.05)

## Discussion

In contrast to the small group of CIS patients analyzed in this study, increased HERVW levels have been associated frequently with MS. Our inability to demonstrate a statistically significant increase of overall HERVW levels in PBMC of CIS patients may be explained by the selection of this particular group or more likely simply by small sample size. However, lack of increased expression is not unprecedented as it was previously reported in a cohort of South African MS patients, although different primers were used for this analysis [17].

We perfomed NGS analysis to identify individual HERVW copies that show altered expression in PBMC, comparing CIS patients (*n* = 5) to controls (*n* = 5). Although more definite answers require future analysis of more subjects, in the CIS patients analyzed more HERVW *loci* are expressed than in control subjects. A similar increase has been reported previously in MS brain [18]. While previous studies failed to identify MS-specific *loci* or expression [18, 19], in the CIS patients we found statistically significant overrepresentation of reads corresponding to specific loci (i.e. 3q11.2 and 19p12, see Table 3 for complete list). Locus-specific qPCR assays may first help confirm this finding in a larger patient cohort, and subsequently be evaluated as a potential prognostic assay.

These combined overrepresented *loci* produce only 1–3 % of total transcripts (Fig. 2 C-E). The combined findings on low levels of overexpression, activation of more *loci*, and activation of low-expressing HERVW elements in CIS patients suggest that their potential contribution to the pathology may be unrelated to overall high expression levels. None of the copies identified encode full-length ENV protein, as the sequences corresponding to the ENV gene are truncated, lack ATG codons, and/or carry frame shifts and STOP codons (Suppl Figure 2). CIS-associated copies may produce proteins (either or not ENV-related) that are especially active in activation of TLR4 [20], or RNAs that trigger the native immune system through TLR3 [21, 22]. Although our analysis shows that upregulation of specific HERVW *loci* in PBMC is associated with CIS, the presence of these transcripts in MS brain is unknown at present. A potential role of these transcripts in proviral protein production and activation of either the peripheral immune system or CNS disease remains to be established.

## Supplementary Information

**Additional file 1: Table S1. **The levels of *GAPDH*, *RPL19* and *HSDA* were determined by qPCR in the samples indicated, as described in the legend to Suppl Fig. 1. The Table lists the Cts obtained for each gene (columns named accordingly, the mean of all three (column “mean”) and the difference between the Cts obtained using either *GAPDH* (G) or the mean of three reference genes (P) (Column P-G). The difference (mean 2.76; standard deviation 0.29) is statistically constant among samples (Welch´s t-test; *p* < 0.05).

**Additional file 2: Table S2. **List of HERVW ENV *loci* according to the GRCh37.p5 version of the human genome database. *Loci* identified by unbiased read mapping were verified as HERVW loci by comparison with the Repbase Update library of repeats from the Genetic Information Research Institute (GIRI)[23], using the the RepeatMasker program. Nomenclature used in Tables is indicated, the genomic location of each copy as well as alternative names used in the literature. Sequences corresponding to the coordinates listed were downloaded and compared to primer sequences using the “align” function in SerialCloner (version 2-6-1). Identity with primer sequence is indicated in black, mismatches in red.

**Additional file 3: Table S3.** Number of reads mapping to the HERVW copies indicated in each of the five CIS or NDI (control) samples. The total number of reads per sample is indicated. The sum of reads in the CIS and NDI groups is indicated.

**Additional file 4: Table S4.** Primers used.

**Additional file 5: Figure S1.** Comparison of reference genes.

**Additional file 6: Figure S2.** Comparison of HERVW elements.

**Additional file 7: Figure S3.** qPCR primer efficiency standard curve analysis.

**Additional file 8: Supplementary Methods.**

**Additional file 9.** MIQE checklist.

## Data Availability

The datasets used and/or analyzed in this study have been deposited in NCBI's Gene Expression Omnibus (Edgar et al., 2002) and are accessible through GEO Series accession number GSE173929 (https://www.ncbi.nlm.nih.gov/geo/query/acc.cgi?acc=GSE173929)
