# Correction to: Upregulation of selected HERVW loci in multiple sclerosis

**DOI:** 10.1186/s13100-021-00247-x

**Published:** 2021-08-11

**Authors:** Sofía Macías-Redondo, Mark Strunk, Alberto Cebollada-Solanas, José-Ramón Ara, Jesús Martín, Jon Schoorlemmer

**Affiliations:** 1 Instituto Aragonés de Ciencias de La Salud (IACS), c/Juan Bosco 13, 50009 Zaragoza, Spain; 2 Sequencing and Functional Genomics, Aragon Biomedical Research Center (CIBA), Instituto Aragonés de Ciencias de La Salud (IACS), Zaragoza, Spain; 3 Aragon Biomedical Research Center (CIBA), Instituto Aragonés de Ciencias de La Salud (IACS), Unidad de Biocomputación, Zaragoza, Spain; 4 Instituto de Investigación Sanitaria de Aragón (IIS Aragón), Zaragoza, Spain; 5 Department of Neurology, University Hospital Miguel Servet, Zaragoza, Spain; 6 ARAID Foundation, Avda. de Ranillas 1-D, 50018 Zaragoza, Spain; 7 Placental Pathophysiology and Fetal Programming Research Group del IISA, c/Juan Bosco 13, 50009 Zaragoza, Spain

**Correction to: Mob DNA 12, 18 (2021)**

**https://doi.org/10.1186/s13100-021-00243-1**

Following the publication of the original article [1] the authors noticed the published Table 1 has a distorted headings and columns not in proper order.

**Table 1:**
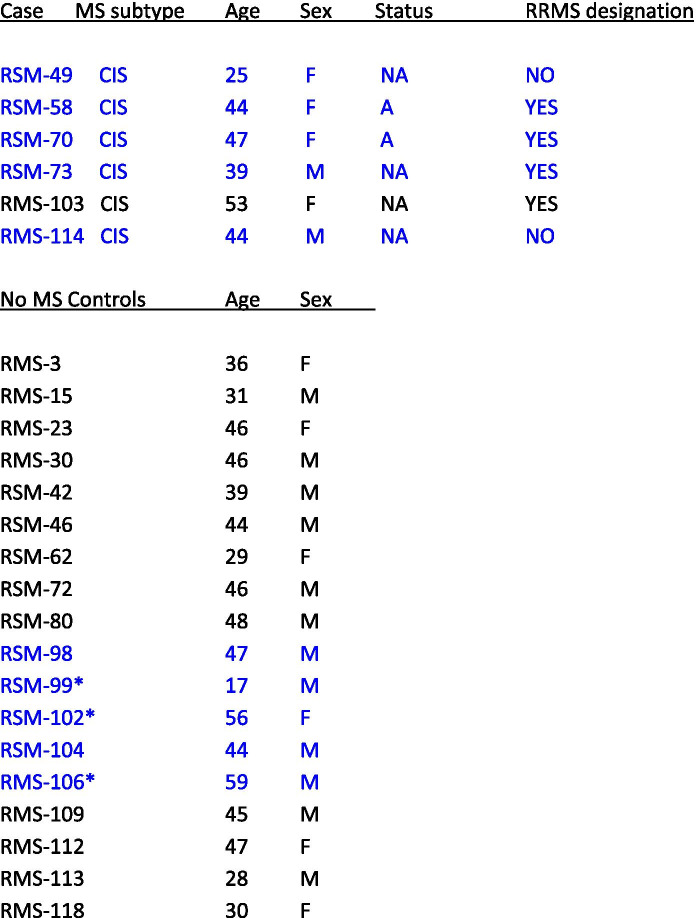
Clinical features of MS patients included in this study

Clinical data of patients whose PBMCs were analyzed for HERVW expression. Median ages for both patients and controles groups were 44 years (mean and SEM are 42,0 ± 4,25 and 40,4 ± 1,94 for patient and control groups, respectively)

A/NA status refers to active and non-active patients respectively. Posterior progression towards MS diagnosis (RRMS) is indicated for all CIS cases. Samples analyzed by NGS are marked in blue. median ages in these groups are 44 year for patients and 47 years for controls. * indicates samples only analyzed by NGS

The correct Table 1 is shown below. The original article [1] has been updated.
