# Supplementary material for: Upregulation of selected HERVW loci in multiple sclerosis
Source: Mob DNA. 2021 Jun 29;12:18. doi: 10.1186/s13100-021-00243-1 (PMC8243764; doi:10.1186/s13100-021-00243-1)
Supplement: Supplementary file 4 — Additional file 4: Table S4. Primers used. [file 13100_2021_243_MOESM4_ESM.docx]

### Table S4

### Supplementary materials

| **Target** | **Sequence (5’-3’)** |
| --- | --- |
| **MSRV *ENV***  **(Mameli et al., 2009)** | For: CTTCCAGAATTGAAGCTGTAAAGC  Rev: GGGTTGTGCAGTTGAGATTTCC  probe: FAM-5’-TTCTTCAAATGGAGCCCCAGATGCAG-3’-TAMRA |
| ***hGAPDH*** | For: ATCAGCAATGCCTCCTGCAC  Rev: TGGCATGGACTGTGGTCATG |

**List of set of primers used for the qPCR expression analysis**. The first column shows the target, the second column indicates the oligo sequence (For= forward; Rev= reverse).
