# Supplementary material for: Upregulation of selected HERVW loci in multiple sclerosis
Source: Mob DNA. 2021 Jun 29;12:18. doi: 10.1186/s13100-021-00243-1 (PMC8243764; doi:10.1186/s13100-021-00243-1)

**Supplementary Figure 1: Comparison of reference genes.**

Levels of *GAPDH*, *RPL19* and *HSDA* were determined by qPCR in cDNAs from all samples included in Figure 1. Cts obtained are depicted on the y axis, each sample tested on the x axis.

Methods: assays were carried out under standard qPCR conditions and requirements described in M&M using the *GAPDH* primers listed (Table S4) and the following Prime Time qPCR assays (IDT technologies):

Hs.PT.58.40170798 HEX/ZEN/IBFQ (SDHA, NM_004168) and

Hs.PT.58.20491224 FAM/ZEN/IBFQ (RPL19, NM_000981)


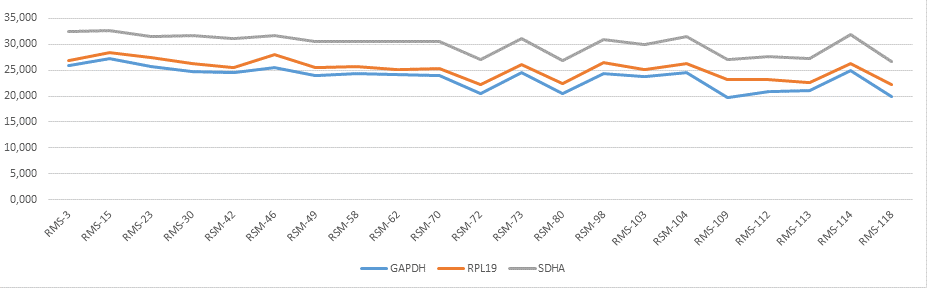

Supplement: Supplementary file 5 — Additional file 5: Figure S1. Comparison of reference genes. [file 13100_2021_243_MOESM5_ESM.docx]
