# Supplementary material for: Upregulation of selected HERVW loci in multiple sclerosis
Source: Mob DNA. 2021 Jun 29;12:18. doi: 10.1186/s13100-021-00243-1 (PMC8243764; doi:10.1186/s13100-021-00243-1)
Supplement: Supplementary file 6 — Additional file 6: Figure S2. Comparison of HERVW elements. [file 13100_2021_243_MOESM6_ESM.pptx]

## Slide 1
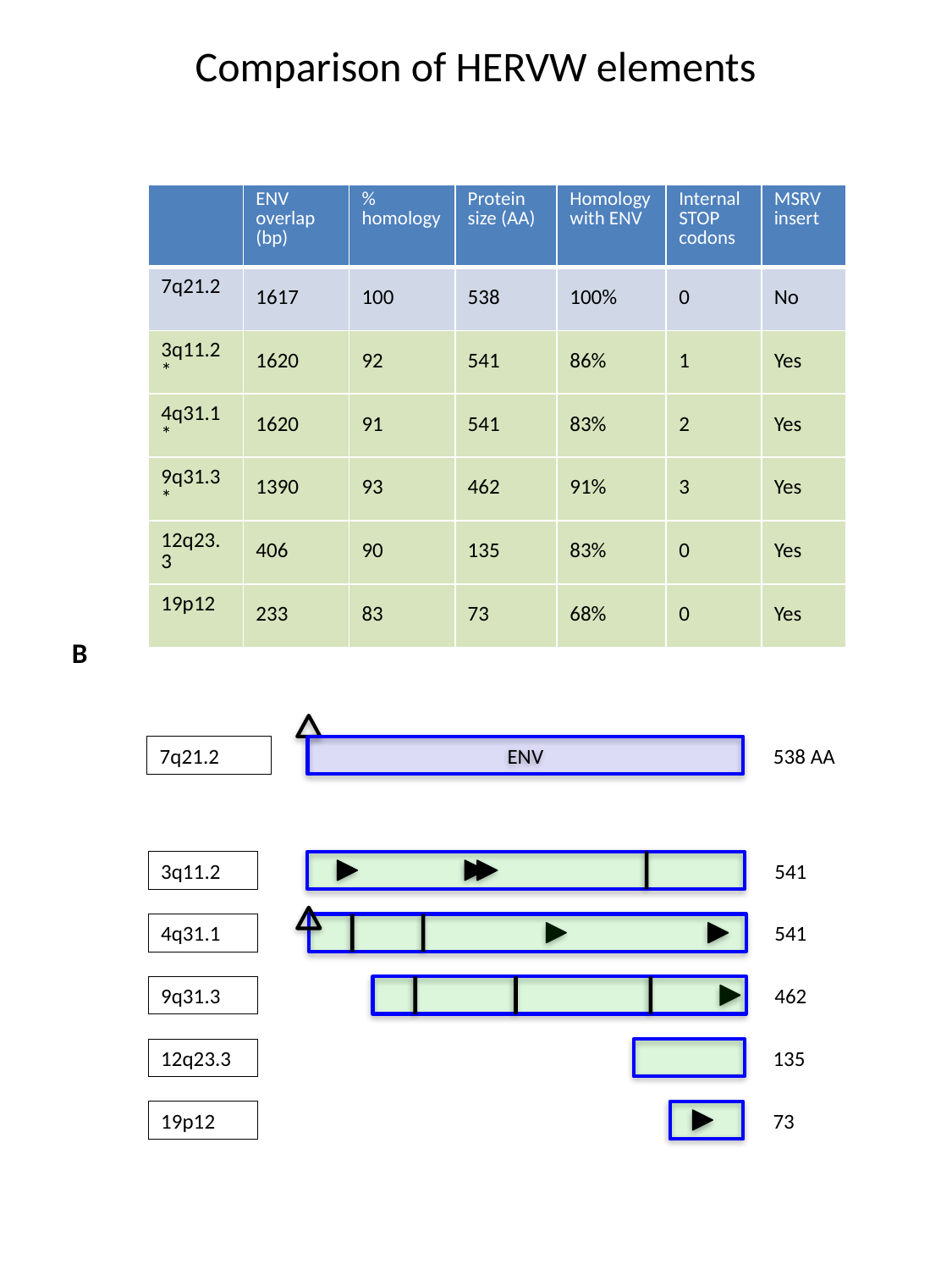

# Comparison of HERVW elements
A
| | ENV overlap (bp) | % homology | Protein size (AA) | Homology with ENV | Internal STOP codons | MSRV insert |
| --- | --- | --- | --- | --- | --- | --- |
| 7q21.2 | 1617 | 100 | 538 | 100% | 0 | No |
| 3q11.2\* | 1620 | 92 | 541 | 86% | 1 | Yes |
| 4q31.1\* | 1620 | 91 | 541 | 83% | 2 | Yes |
| 9q31.3\* | 1390 | 93 | 462 | 91% | 3 | Yes |
| 12q23.3 | 406 | 90 | 135 | 83% | 0 | Yes |
| 19p12 | 233 | 83 | 73 | 68% | 0 | Yes |
 B
538 AA
7q21.2
ENV
3q11.2
541
4q31.1
541
462
9q31.3
135
12q23.3
19p12
73
