# Supplementary material for: Upregulation of selected HERVW loci in multiple sclerosis
Source: Mob DNA. 2021 Jun 29;12:18. doi: 10.1186/s13100-021-00243-1 (PMC8243764; doi:10.1186/s13100-021-00243-1)

**Suppl Figure 3: qPCR primer efficiency standard curve analysis.** A qPCR standard curve is graphically represented as a semi-log regression line plot of C_t_ value versus log of input cDNA. The efficiency (E) is calculated with the formula E=10 ^(-1/slope)^-1. Thus, a slope of -3.32 indicates a PCR reaction with 100% efficiency. A range of 90% < E < 110% was considered acceptable. Efficiency is regular over the C_t_ interval shown, which therefore also indicates the lower limit of detection.

MSRVenv refers to the HERVW assay [13] used for Figure 1.


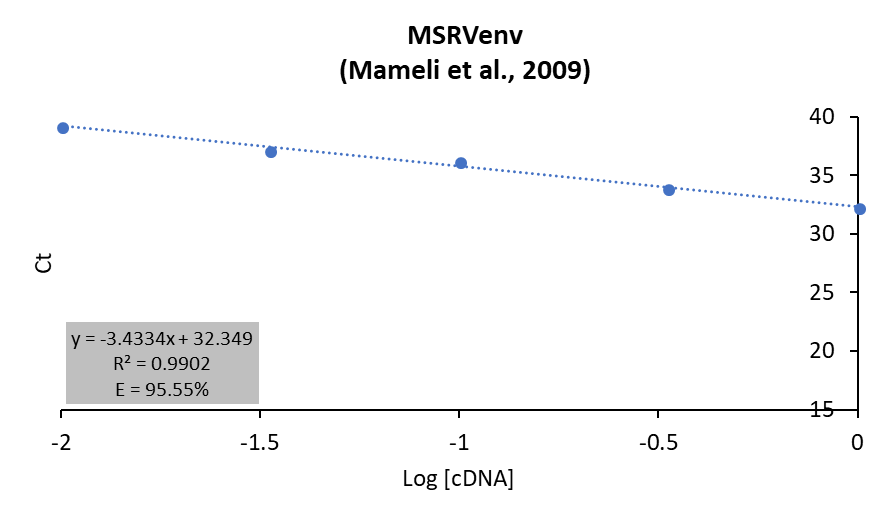


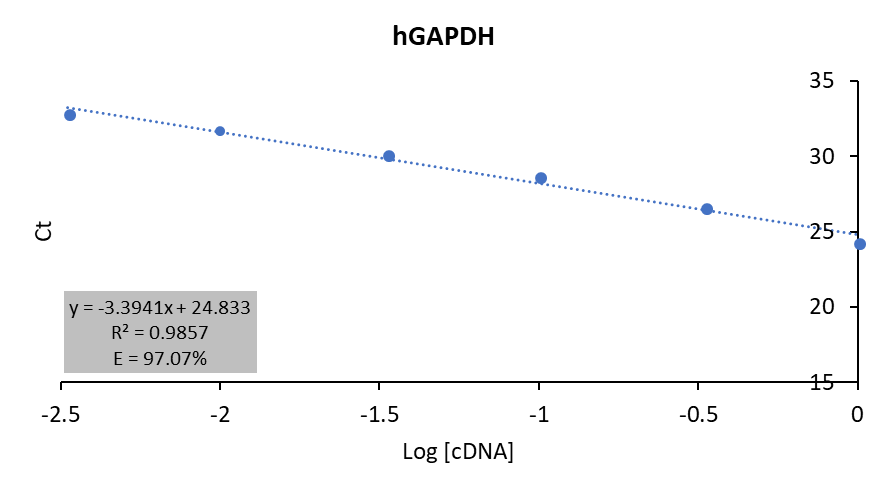

Supplement: Supplementary file 7 — Additional file 7: Figure S3. qPCR primer efficiency standard curve analysis. [file 13100_2021_243_MOESM7_ESM.docx]
