# Supplementary material for: Upregulation of selected HERVW loci in multiple sclerosis
Source: Mob DNA. 2021 Jun 29;12:18. doi: 10.1186/s13100-021-00243-1 (PMC8243764; doi:10.1186/s13100-021-00243-1)
Supplement: Supplementary file 8 — Additional file 8: Supplementary Methods. [file 13100_2021_243_MOESM8_ESM.docx]

**Supplementary methods**

**Study subjects.**

Blood samples were collected from MS patients and healthy controls from the Neurology Department of Miguel Servet University Hospital (Zaragoza, Spain). Whole fresh blood was drawn into vacutainer tubes (Becton Dickinson Vacutainer) containing EDTA. Within 24 hours, peripheral blood mononuclear cells (PBMC) were isolated using Histopaque-1077 (Sigma) as previously described[12].

**Quantitative PCR**

PBMCs were homogenized in TRIzol® reagent (Invitrogen) and frozen at -80ºC. Total RNA was subsequently isolated from the resulting homogenate using an in-house method [13]. Isolated RNA was quantified using a Nanodrop spectrophoto-meter (Thermo Scientific, Nanodrop 2000) and kept at -80ºC until further use. Regular quality control is performed in the laboratory to ensure RNA isolation from fresh tissue or cells in Trizol yields high quality RNA (RIN values ≥ 8). Starting from 500 ng of RNA, cDNA was synthesized with random hexamer primer using the ThermoScriptTM RT-PCR System (11146-024, Invitrogen) according to the manufacturer’s protocol.

Gene expression was analyzed by Real-Time qPCR on a ViiA™ 7 Real-Time PCR System (Applied Biosystems), using 1 μl of 10 fold diluted cDNA and the following thermal cycling conditions: [50°C, 2’ - 95°C, 10’ - (95°C, 15’’ - 60°C, 1’)x40 - 95°C, 15’’ - 60°C, 20’’ - 95°C, 15’’]. HERVW *ENV* expression analysis was performed in a probe-based qPCR assay using a specific pair of primers and probe (PrimeTime qPCR Probes; Integrated DNA Technologies, Inc.) for MSRV *ENV* [15], using the Premix Ex TaqTM Master Mix (RR390A, Takara). Analysis of *hGAPDH* expression was performed using standard SYBR Green based detection (SYBR Premix Ex Taq II, RR820A, Takara). Melting curves were verified for production of a single DNA species. Sequences of all primers used are described in Table S4. Reactions (15 μl) were carried out using primer, probe and dNTP concentrations specified by the provider (Takara Bio Inc).

Amplification efficiency of all set of primers between 90% and 110% was assessed in calibration curves (Suppl. Figure 3). All reactions were carried out in triplicate and only measurements with a standard deviation < 0.2 were considered. Target gene expression levels were recalculated as 2^-ΔΔCt^ with respect to the median ΔCt value of the samples in the control group, using *hGAPDH* as a reference gene [13]. Procedures and reporting requirements mostly follow MIQE guidelines. A completed MIQE checklist is provided as Suppl Material (The MIQE Guidelines: Minimum Information for Publication of Quantitative Real-Time PCR Experiments. Bustin et al., Clinical Chemistry 55:4 611-622 (2009).

**Amplification products for NGS**

For the identification and localization of transcribed HERVW *loci*, cDNA was amplified employing the external primers of an established probe-based PCR assay for HERVW ENV [15] in a 50-µl volume using 2 µl of cDNA, 400 nM each primer), 200 µM dNTPs and 0.5 U Taq DNA Polymerase (D1806, Sigma-Aldrich). PCR cycling conditions were as follows: initial activation at 98 ºC for 2 min, followed by 35 cycles of 95 ºC for 30 seconds, 60 ºC for 30 seconds and 72 ºC for 30 seconds and 10 minutes extension at 72 ºC. PCR products were purified using Diffinity RapidTip®2 (D2947, Sigma).

**Next Generation Sequencing analysis**

Library preparation and sequencing was carried out using the workflow and kits approved for IonTorrent technology (Beckman Coulter, Thermofisher, Kapa/Roche). NGS was performed on a Ion Torrent S5XL platform using a Ion 530 chip. After removing barcodes and PCR primer sequences from the resulting Fastq files, reads were mapped to the human reference genome (version GRCh37/hg19) using the Torrent MApping Program (TMAP v5.2.22). Strict criteria were applied to maximize mapping differences between different copies (soft clipping off, maximum mismatch and open gap penalties, gap extension penalty 10, minimum alignment length 135, "unique best hit and stage1 map4"). Relevant HERVW *loci* were identified by unbiased read mapping, verified as HERVW loci using the RepeatMasker program (GRCh37.p5 version of the human genome database), which uses the Repbase Update library of repeats from the Genetic Information Research Institute (GIRI)[23]. For each sample, the number of reads corresponding to individual HERVW copies listed was calculated, as well as the cumulative error rate. We used an ad hoc nomenclature based on chromosomal location (i.e. chr3-1) or alternative names suggested by the RepeatMasker track of the UCSC Genome Browser as indicated in Supplementary Tables.

Relative frequencies were calculated as the number of reads mapping to an individual HERVW ENV element relative to the total number of reads. These frequencies were used to compare the relative expression in cases versus controls (Figure 2B). To calculate fold increase in patients versus controls, the sum of reads mapping to a particular locus was compared per group, after correction for the difference in total reads per group (1,2134722 fold, Table S3).

**Statistical analysis**

SPSS software was used for all analyses (IBM Corp. Released 2013. IBM SPSS Statistics for Windows, Version 15.0). The graphs were generated using SPPS software as well. Normality was assessed with Shapiro-Wilk test (n< 50). To assess the statistical significance of differences, a specific test was performed depending on normality and number of groups of samples compared. Non-normally distributed data in Figure 1 were analyzed applying the U de Mann-Whitney test (2 groups). Normally distributed data in Figure 2A were analyzed using t-student test (2 groups). The significance of the copy-specific frequencies displayed in Table 2 were analyzed using the DESeq2 package [16] to correct p values for multiple testing (False Discovery Rate < 0.05).
